# Cytosolic BolA Plays a Repressive Role in the Tolerance against Excess Iron and MV-Induced Oxidative Stress in Plants

**DOI:** 10.1371/journal.pone.0124887

**Published:** 2015-04-30

**Authors:** Lu Qin, Meihuan Wang, Jia Zuo, Xiangyang Feng, Xuejiao Liang, Zhigeng Wu, Hong Ye

**Affiliations:** 1 Key Laboratory of Plant Resources Conservation and Sustainable Utilization, South China Botanical Garden, Chinese Academy of Sciences, Guangzhou 510650, China; 2 Oil Crops Research Institute, Chinese Academy of Agricultural Sciences/Key Laboratory of Biology and Genetic Improvement of Oil Crops, Ministry of Agriculture, Wuhan 430062, China; Texas Tech University, UNITED STATES

## Abstract

The BolA-like protein is present in all eukaryotes, and it is able to form complex with monothiol glutaredoxin of the same subcellular compartments, suggesting that the BolA-like protein has essential function in eukaryotes, and that the function is associated with its partner glutaredoxin. Some studies have indicated a role for BolA proteins in Fe-S cluster synthesis or in redox homeostasis. However, the physiological function of BolA proteins remains to be elucidated. Here, we report the characterization of an insertion mutant of *BolA3* in Arabidopsis. Among the four AtBolA proteins found in Arabidopsis, the AtBolA3 was the only BolA located in the cytosol of plant cells. It was highly expressed in roots. AtBolA3 was able to interact with the cytosolic monothiol glutaredoxin, AtGRXS17. The *bola3* mutant did not show any notable phenotype under normal growth condition, but rather grew better than wild type under some stresses. The *bola3* mutant was more tolerant to excess iron and the MV-induced oxidative stress than wild type. It displayed no necrosis in leaves, developed longer roots, accumulated more iron and higher Fe-S protein activities in roots. In addition, the mutant possessed a more potent antioxidant defense to scavenge ROS species. Taken together, our data indicated that the cytosolic AtBolA3 has a suppressive role in the tolerance to excess iron and the MV-induced oxidative stress in plants. AtBolA3 seems to be a repressor under some stress conditions.

## Introduction

Iron is an essential micronutrient for plants, which is used for the biosynthesis of heme and iron-sulfur (Fe-S) clusters [1,2,3]. Although iron is abundant in soil, the bioavailability of iron is always limited by high pH values of soil. On the other hand, excess iron could be toxic to plants in acidic soil. Nongraminaceous and graminaceous plants develop two distinct strategies to acquire iron from soil, iron reduction strategy and iron chelation strategy, respectively [4,5,6]. The iron nutrients are transported from the root to the shoot, taken up into plant cells, and distributed into subcellular compartments, where they are used to assemble iron-containing cofactors, in particular, the Fe-S clusters [2,7]. Fe-S proteins have many important physiological functions, such as electron carriers in the electron transfer chains, enzymes in redox reactions, regulatory sensors, and stabilizers of protein structures [1,8]. Despite that Fe-S clusters simply consist of two elements, the biosynthesis of Fe-S clusters is highly complex in the living cells. In plants, for example Arabidopsis, more than 40 genes are thought to facilitate the biogenesis of Fe-S proteins in plastids, mitochondria, cytosol and nucleus [2]. As an activator of NFS2, SufE1 consists of a N-terminal SufE domain and a C-terminal domain with similarity to the bacterial BolA [9].

BolA-like proteins are ubiquitous, and are present in numerous organisms from bacteria to higher eukaryotes. A *BolA* gene was originally identified in *Escherichia coli*. It was able to produce osmotically stable round cells when overexpressed, and this morphology effect of *BolA* might be related to cell division [10]. Further results showed that the expression of *BolA* gene could be induced by different stress conditions, such as osmotic shock and oxidative stress [11]. In the yeast, *Schizosaccharomyces pombe*, a *BolA* ortholog is thought to be a UV-inducible gene and may play a role in cell cycle [12].

Some other studies, however, have indicated that the BolA protein may be involved in the regulation of iron homeostasis. In *Saccharomyces cerevisiae*, glutaredoxin proteins, Grx3 and Grx4, form a complex with Fra1, an aminopeptidase P-like protein, and Fra2, a BolA-like protein [13]. By bridging an internal [2Fe-2S] cluster, the complex senses the status of mitochondrial Fe-S cluster assembly, and regulates the localization of Aft1, a transcription factor. The nuclei-localized Aft1 controls the expression of many genes involved in iron uptake and storage [14,15,16]. In addition, an interaction between glutaredoxin (Grx) and BolA proteins is also found in *Drosophila melanogaster*, *Arabidopsis thaliana*, and *Homo sapiens* [17,18,19,20,21,22], suggesting that the Grx-BolA interaction is a universal phenomenon in eukaryotes.

BolA proteins are identified in humans. Study on a human patient with a mutation in the *BolA3* gene revealed that *BolA3* encodes a mitochondrial protein that plays an essential role in the maturation of Fe-S centers in the lipoate-containing 2-oxoacid dehydrogenases and in the respiratory chain complexes [23]. Another mitochondrial BOLA1 protein in humans could prevent mitochondrial morphology aberrations induced by glutathione (GSH) depletion, and reduce the associated oxidative shift of the mitochondrial thiol redox potential. BOLA1 forms a complex with GLRX5 in human cells [22]. In Arabidopsis, four BolA-like proteins have been identified, including SufE1. They are distributed in plastids, mitochondria, and cytosol, respectively. These BolA-like proteins can interact with all monothiol glutaredoxins of the same subcellular compartment, and this interaction may allow the activity of the BolA protein to be redox regulated by glutaredoxin [19]. However, it still remains unknown regarding the physiological function of a BolA-like protein in plants.

In this study, we have identified four *BolA*-like genes in Arabidopsis. One of the genes, which we named *AtBolA3*, encodes a protein with a high similarity to the BolA3 in humans. AtBolA3 was located in cytosol, and it was highly expressed in roots. We have characterized the homozygous *bola3* insertion mutant plants. Data showed that AtBolA3 was not required for the maturation of typical Fe-S proteins, and that the mutant plants grew normally. However, we found that the *bola3* mutant was more resistant to excess iron and the MV-induced oxidative stress than wild type. The mutant accumulated more iron and higher Fe-S protein activities in roots under the stresses. The *in vivo* study suggested that AtBolA3 may function as a repressor in the resistance to excess iron and oxidative stress in roots, likely by interacting with AtGRXS17, a cytosolic glutaredoxin. Our findings on the cytosolic BolA in plants provide insight into the function of BolA-like proteins.

## Materials and Methods

### Multiple sequence alignment

Homologous BolA proteins were retrieved from Arabidopsis genome database (TAIR, http://www.arabidopsis.org), using the sequence of either HsBolA3 or ScFra2 as query in BlastP search. Alignment of amino acid sequences was done with ClustalX, and visualized with Genedoc software. The conserved domain structure of proteins was drawn with tools in NCBI (http://www.ncbi.nlm.nih.gov). Gene nomenclature was recommended by Botanical Institute in University of Cologne (http://www.botanik.uni-koeln.de). The subcellular localization of proteins was predicted with the WoLF-PSORT and Target P tools.

### Plant materials and growth conditions

*Arabidopsis thaliana* (L.) Heynh of Columbia-0 (Col-0) was used as the wild type, which is also the genetic background of mutant plants in this study. Seeds of the T-DNA insertion line of *bola3* (SALK_013477) were obtained from the Salk Institute (http://signal.salk.edu) via the Arabidopsis Biological Resource Center (ABRC). In petri dish culture, seeds were surface sterilized and germinated on half-strength Murashige and Skoog (MS) agar medium supplemented with 1.5% sucrose, after stratification at 4°C for 3 days. In hydroponic culture, seedlings were transferred to the nutrient solution for Arabidopsis after germination on vermiculite for 7 to 10 days. The growth condition was 22°C and a 16 hours/8 hours (light/dark) cycle. Nutrients in the hydroponic solution included 2 mM Ca(NO_3_)_2_, 5 mM KNO_3_, 1 mM KH_2_PO_4_, 2 mM MgSO_4_, 50 μM Fe-EDTA, 70 μM H_3_BO_3_, 1 μM ZnSO_4_, 14 μM MnCl_2•_4H_2_O, 0.5 μM CuSO_4•_5H_2_O, 0.2 μM Na_2_MoO_4_ and 0.01 μM CoCl_2•_6H_2_O.

### Plasmid construction and plant transformation

To generate construct for the subcellular localization analysis, 282 bp of coding region of *AtBolA3* was amplified with primers shown in S1 Table. After digestion with *Sal*I and *Sac*I, the coding region of *AtBolA3* was fused to the p-35S-GFP vector driven by a CaMV 35S promoter. To generate construct for the promoter analysis, 2 kb of the putative promoter region of *AtBolA3* was amplified with primers shown in S1 Table. After digestion with *Hind*III and *Pst*I, the promoter region of *AtBolA3* (AtBolA3prom) was fused to the pCAMBIA1391z vector (CAMBIA) containing β-glucuronidase (GUS) reporter gene. The resulting AtBolA3prom::GUS construct was introduced into *Agrobacterium tumefaciens* GV3101, which was used to transform Arabidopsis through the floral dip method [24]. To generate construct for genetic complementation of *bola3* mutant, the coding region of *AtBolA3* was cloned into the plant expression vector pBA002-3HA. The resulting construct was used to transform the homozygous *bola3* mutant through floral dip. In bimolecular fluorescence complementation (BiFC) analysis, the coding sequences of *AtBolA3* and two candidate genes, *AtGRXS17* and *AtAPP1*, were amplified with primers listed in S1 Table. The *AtBolA3* sequence was cloned into vector pSAT6-cEYFP-C1, while *AtGRXS17* or *AtAPP1* sequence was cloned into vector pSAT6-nEYFP-C1.

### Identification of *bola3* knockout mutant

Mutant lines of *Arabidopsis thaliana* (Columbia-0 ecotype) containing random T-DNA insertions were screened using PCR as described previously [25]. The *AtBolA3* specific primers and a T-DNA specific primer (S1 Table) were used in PCR to detect the insertion in *AtBolA3* gene. Transcripts of *AtBolA3* in mutants were verified by both semi-quantitative RT-PCR and quantitative RT-PCR.

### Iron and methyl viologen (MV) treatments

Seeds were germinated on vermiculite for 7–10 days, and transferred to nutrient solution. At 4 weeks of age, plants were treated with either excess iron or methyl viologen (MV). After treatment in 500 μM Fe-EDTA for 4 weeks, plants were harvested for subsequent measurement of main root length and iron concentration, and the Fe-S enzyme activity assays. After treatment in 50 μM MV for 0.5 hour, 2 hours and 12 hours, plants were harvested for the photograph of phenotypes, antioxidant enzyme assays, and gene expression analysis. After treatment in 2 μM MV for 4 weeks, plants were harvested for iron concentration measurement and Fe-S enzyme assays. In the MV treatment on Petri dishes, seeds were germinated and grown on half-strength MS agar medium containing 0.15 μM MV for 2 weeks.

### RNA extraction, semi-quantitative RT-PCR and quantitative RT-PCR

Total RNA was extracted from Arabidopsis plants using Plant RNA Extraction Kit (Omega, Norcross, GA, USA). The first-strand complementary DNA was synthesized using reverse transcription kit (TaKaRa, Kyoto, Japan). Gene specific primers were listed in S1 Table. In RT-PCR, 1 μL of cDNA was used in a 20 μL PCR reaction. The PCR program for the gene expression analysis was 2 min denaturation at 94°C, followed by 30 cycles of the following steps: 94°C for 30 s, 57°C for 30 s, 72°C for 30 s. In quantitative RT-PCR (qRT-PCR), the reaction was carried out in a 20 μL volume containing 2 μL of 1:5 diluted complementary DNA, 0.2 mM primers, and 10 μL of SYBR Premix Ex Taq (TaKaRa, Kyoto, Japan). All reactions were carried out on a Light Cycler 480II (Roche, Basel, Switzerland). The thermal cycling program was: 95°C for 1 min, 40 cycles of 95°C for 15 s, (54–62)°C for 15 s and 72°C for 30 s. Fluorescence data were collected at 72°C during the cycles. Relative gene expression was calculated as the ratio of the expression of target gene to that of a housekeeping gene, *AtUBQ11*, with four independent biological replicates.

### Histochemical analysis of GUS expression

7-day-old transgenic seedlings of the T3 generation were incubated in the GUS staining solution (0.2 M Na_2_HPO_4_-NaH_2_PO_4_ buffer, pH 7.0, and 1 mM 5-bromo-4-chloro-3-indolyl-b-D-glucuronic acid) at 37°C for 24 h [26]. The stained samples were then washed with 50 mM sodium phosphate buffer, pH 7.0, and cleared in 70% (v/v) ethanol. The histochemical staining of GUS was visualized under a light stereomicroscope (LEICA DFC420, Germany).

### Enzyme activity assays

Proteins were extracted by grinding 200 mg of leaf tissues or 50–100 mg of root tissues in an equal volume of extraction buffer (50 mM Tris-HCl, pH 8.0, 50 mM NaCl, 1% (v/v) Triton X-100, 1 mM dithiothreitol, Roche’s protease inhibitor cocktail, 1 mM PMSF), followed by centrifugation at 12,000 rpm, 4°C for 30 min. The supernatant was mixed with 0.25 volume of loading buffer (20 mM Tris-HCl, pH 8.0, 80% [v/v] glycerol, and 0.1% [w/v] bromphenol blue), and separated on Native PAGE gels for four hours. The gels were then incubated in staining solutions containing substrates for aconitase (ACO), aldehyde oxidase (AO), and xanthine dehydrogenase (XDH), respectively. Details about the in-gel activity assays for ACO, AO and XDH were described previously [27,28]. Equal loading was evaluated by Coomassie blue staining of gels as shown in S1 Fig. Two biological replicates per genotype were used. The in-gel activities of ACO, AO and XDH were quantified using Image J software (NIH, USA).

In the antioxidant enzyme assays, the leaf or root tissues were homogenized in the ice-cold phosphate-buffered solution (PBS) (50 mM, pH 7.0), followed by centrifugation at 12,000 rpm, 4°C for 30 min. The supernatant was immediately used to analyze the activities of SOD, POD, and CAT enzymes. The SOD activity was assayed by monitoring the 560 nm absorbance using the nitro-blue-tetrazolium (NBT) reduction method [29]. POD activity was determined by monitoring the 470 nm absorbance using the guaiacol method [30]. CAT activity was measured by following the decrease in absorbance at 240 nm using the ultraviolet absorbance method with the addition of H_2_O_2_. Protein concentration was determined using Bio-Rad protein assay reagent. Four biological replicates were used for each measurement.

### Detection of reactive oxygen species (ROS)

7-day-old wild type and *bola3* mutant seedlings were placed in hydroponic culture containing 2 μM MV for 2 hours. Superoxide accumulation was visualized using the NBT staining method as described [31]. Briefly, the seedlings were immediately incubated in 0.5 mg/mL NBT in 10 mM potassium phosphate buffer, pH 7.6, in dark at 25°C for 2 hours. Each measurement was repeated with at least five biological replicates. The level of ROS staining was quantified using Image J software (NIH, USA).

### Iron quantification

Arabidopsis seedlings were washed with distilled water, fixed at 105°C for 30 minutes, and dried in an oven at 75°C, followed by the measurement of dry weights. The samples were ground and digested in 5 mL of concentrated HNO_3_ overnight, and digested on Multiwave 3000 (Anton Paar) for 2 hours. Iron was quantified on ICP-MS 7700 series (Agilent Technologies). Scandium was used as an internal standard. Three biological replicates were used for each measurement.

### Subcellular localization and BiFC analysis in Arabidopsis protoplasts

Protoplasts were isolated from the leaves of 4-week-old Arabidopsis plants (Columbia ecotype), according to Sheen’s protocol (http://genetics.mgh.harvard.edu). About 2*10^5^ protoplasts were transfected with 20 mg plasmid DNA via polyethylene glycol 4000, incubated in a plate under weak light for 12–16 hours, and then observed in a Leica TCS SP5 laser scanning confocal microscope (LEICA, Germany).

### Accession Numbers

Sequence data for this article can be found in Arabidopsis Genome Initiative data libraries under the following accession numbers: AtBolA1 (At4g26500), AtBolA2 (At1g55805), AtBolA3 (At5g09830), AtBolA4 (At5g17560), AtUBQ11 (At4g05050), AtCSD1 (At1g08830), AtCAT2 (At4g35090), AtGRXS17 (At4g04950).

## Results

### Identification of *BolA*-like genes in Arabidopsis

**Fig 1:**
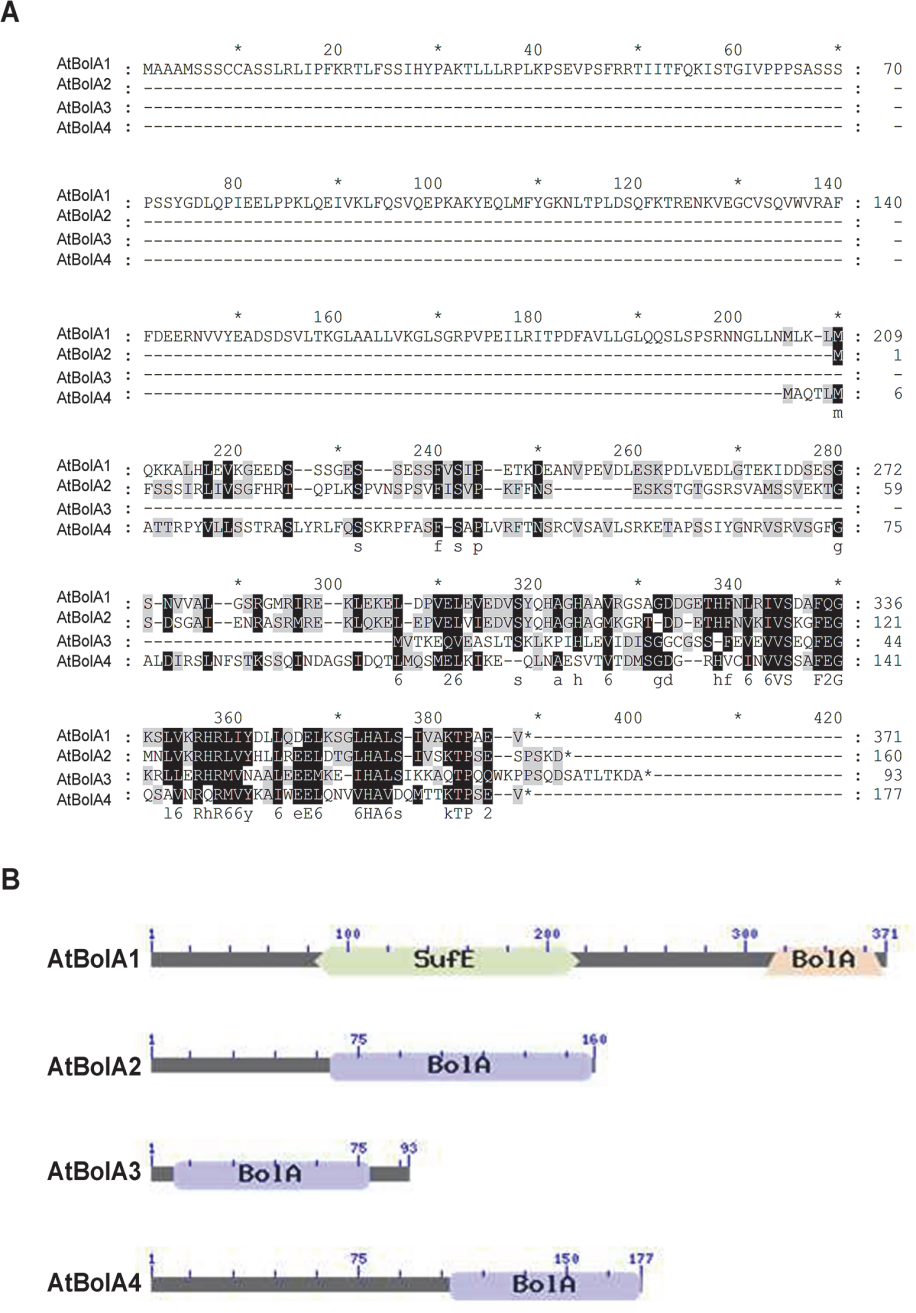
Identification of four BolA-like proteins in *Arabidopsis thaliana*. (A) Multiple sequence alignment of amino acid sequences of AtBolA1, AtBolA2, AtBolA3, and AtBolA4. (B) Conserved domains of BolA-like proteins in *Arabidopsis thaliana*. The conserved domain architecture was drawn using the tool in NCBI website (http://www.ncbi.nlm.nih.gov).

Using the sequence of human HsBolA3 as query, BolA homologs were retrieved from Arabidopsis genome database (TAIR). A total of four BolA homologs were identified in Arabidopsis (Fig 1). According to the recommended nomenclature, these genes were named as *AtBolA1* through *AtBolA4* respectively, in an order corresponding to their chromosome locations. Protein sequence alignment was performed to analyze the similarity (Fig 1A). Amino acids were highly conserved in the C-terminus of proteins, which was annotated as the BolA domain (Fig 1B). In the BolA domain, a motif of SxxF(x18)E(x5)H was conserved in all BolA-like proteins from dozens of species, including microbes, plants, and animals (not shown). By analog to the residue in BolA-like proteins of yeast and humans [16,17], the histidine residue in the highly conserved motif is likely required to covalently chelate an iron of the [2Fe-2S] cluster. AtBolA1 is also named AtSufE1 as described previously [9]. AtBolA2, 3, 4 in our nomenclature correspond to BolA1, 2, 4 in a previous report [19]. Notably, the AtBolA3 protein (Accession number: At5g09830) has an identity of 53% to the HsBolA3, which is thought to be involved in Fe-S cluster biosynthesis and iron homeostasis in mitochondria [23].

### Subcellular localization and tissue specific expression of AtBolA3

**Fig 2:**
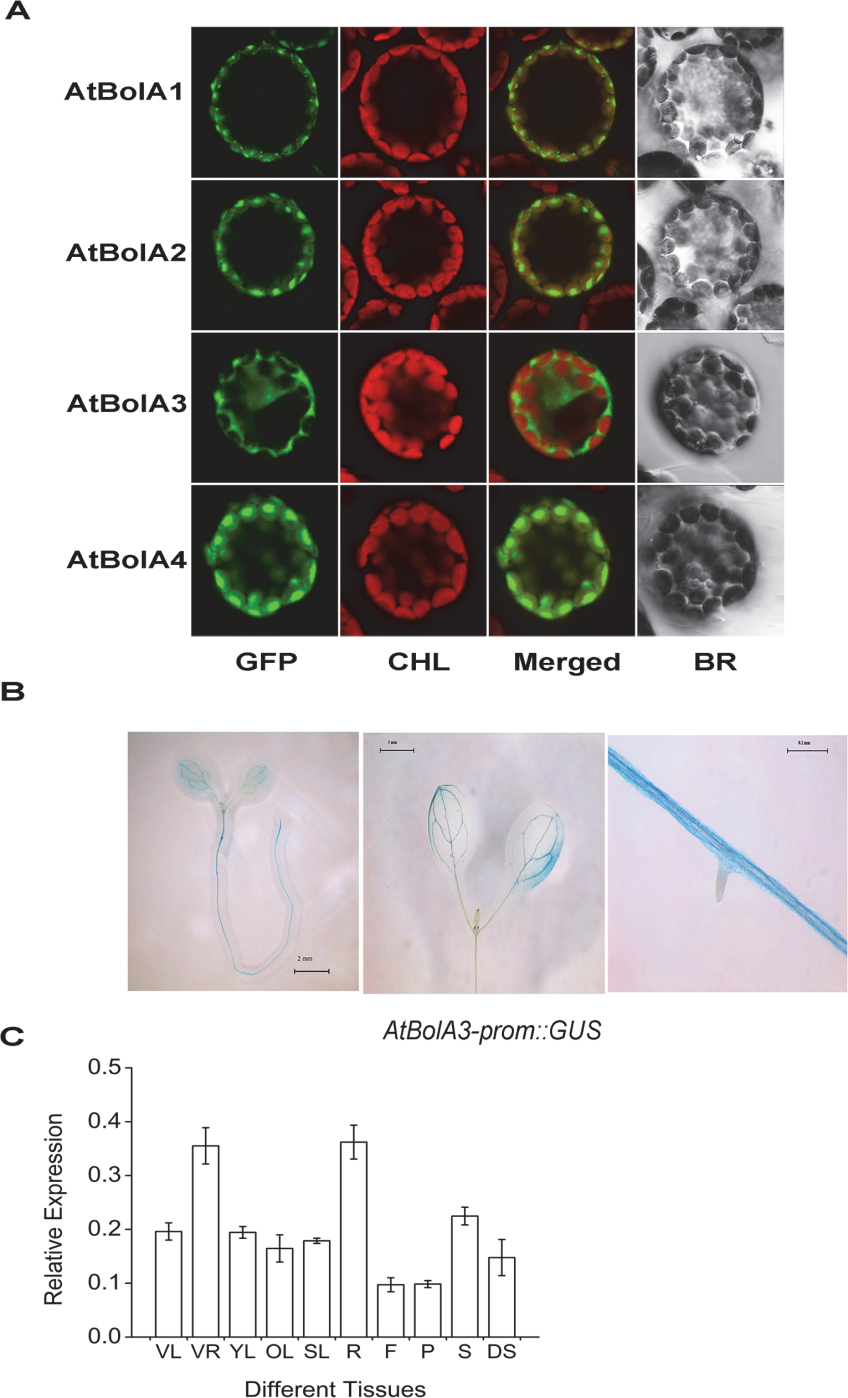
Subcellular localization of four AtBolA proteins and tissue specific expression of *AtBolA3* in *Arabidopsis thaliana*. (A) Subcellular localization of BolA1, BolA2, BolA3 and BolA4 in Arabidopsis protoplasts, which were transiently transformed with BolA-GFP constructs. CHL: chloroplast; BR: bright field; Merged: GFP and CHL overlay. (B) Spatial expression pattern of AtBolA3prom::GUS in one-week-old seedlings under normal growth condition. (C) Expression of *AtBolA3* detected by qRT-PCR in various tissues of *Arabidopsis thaliana* at the vegetative and reproductive stages. The relative expression was calculated as the ratio of the expression of *AtBolA3* to that of housekeeping gene, *AtUBQ11*, with four independent biological replicates. VL and VR: leaf and root in the vegetative stage; YL, OL, SL, R, F, P, and S stand for young leaf, rosette leaf, stem leaf, root, flower, pod, seed in the reproductive stage. DS: dry seed.

As predicted by WoLF-PSORT and TargetP tools, three BolA (AtBolA1, 2, and 4) were located in chloroplasts, whereas AtBolA3 was in cytosol. The predicted subcellular localization was experimentally examined by transient expression of GFP fusion proteins in Arabidopsis mesophyll protoplasts. The N-terminal targeting peptides lead the fusion proteins into specific subcellular compartments. The GFP fluorescence indicated that AtBolA1 and AtBolA2 proteins were indeed in chloroplast (Fig 2A), and the vast majority of AtBolA4 was also in chloroplast. Only the AtBolA3 was cytosolic. The unique localization of AtBolA3 in cytosol and a high similarity to both HsBolA3 and ScFra2, which are thought to be involved in iron homeostasis, prompted us to further investigate AtBolA3.

To analyze the histological expression of *AtBolA3*, we generated AtBolA3prom::GUS transgenic plants. GUS expression was detected in both shoots and roots of the one-week-old AtBolA3prom::GUS seedlings (Fig 2B). Strong GUS staining was observed in epidermal and cortex cells of roots, whereas staining was observed in the veins of leaves. Next, we performed quantitative RT-PCR (qRT-PCR) to analyze *AtBolA3* expression in all major tissues (Fig 2C). Although this gene was expressed in all tissues, the expression in roots was about 2-fold higher than other tissues at both vegetative and reproductive growth stages (Fig 2C).

### Characterization of the *bola3* insertion mutant

**Fig 3:**
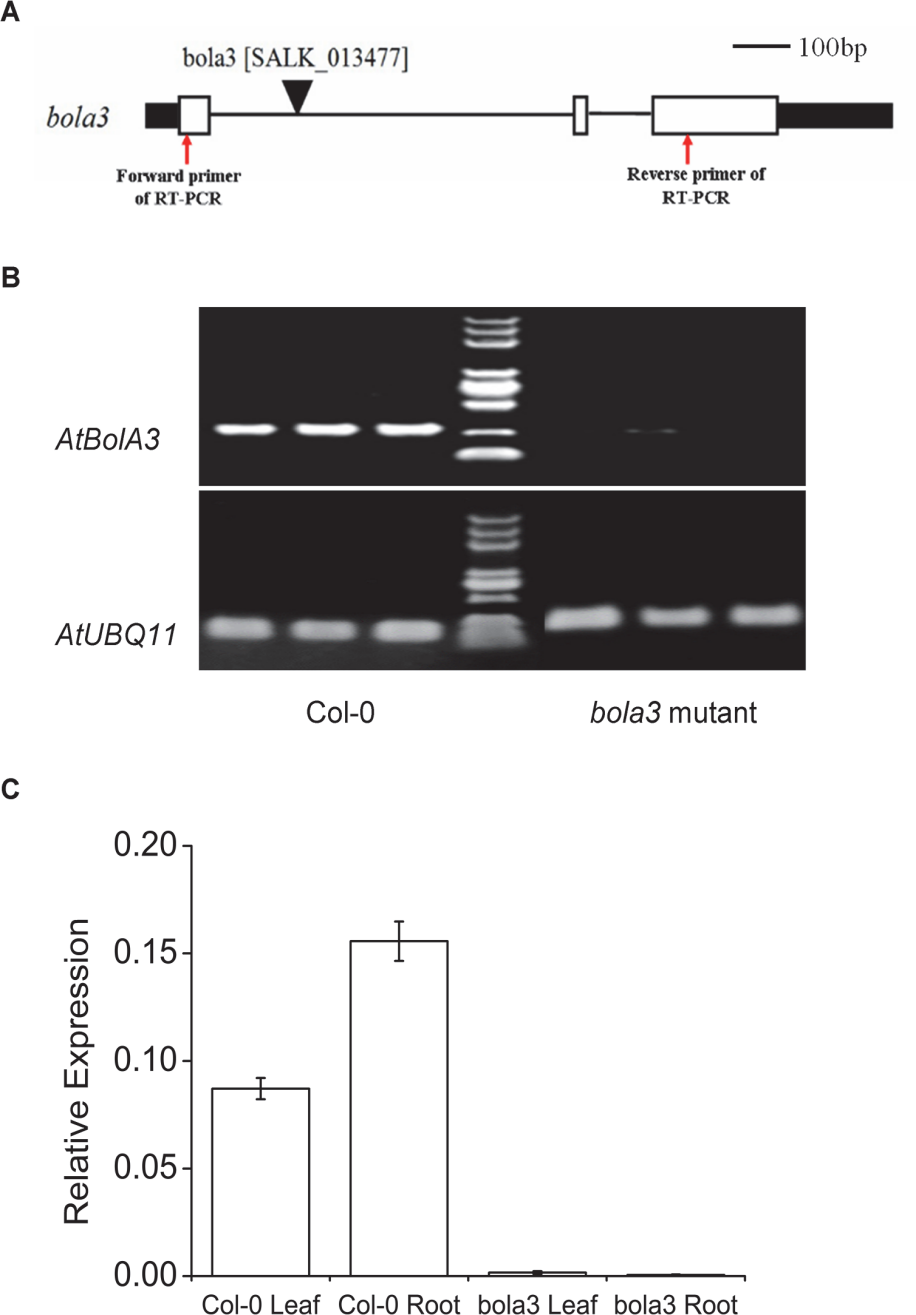
Identification of the *bola3* insertion mutant. (A) Gene structure of *AtBolA3*. The positions of T-DNA insertion and primers used in RT-PCR were marked in the diagram. Black bar: UTR; White bar: exon. Bar = 100bp. (B) Analysis of *AtBolA3* gene expression in Col-0 and *bola3* mutant by semi-qRT-PCR. The housekeeping gene, *AtUBQ11*, was used as internal control. (C) Analysis of *AtBolA3* gene expression in the leaf and root of Col-0 and *bola3* mutant by qRT-PCR with four independent biological replicates.

To investigate the function of AtBolA3 in Arabidopsis, two insertion mutant lines were obtained from the Arabidopsis Biological Resource Center. Homozygous mutant plants were screened from one line (SALK_013477), whereas the other line (SALK_254B03) turned out to be all wild type. The homozygous mutant line (SALK_013477) was used for subsequent characterization. This mutant line contains a T-DNA insertion at the intron-1 (Fig 3A), which interferes with the full length mRNA transcription and normal splicing, therefore reducing the amount of mature *AtBolA3* transcript. In homozygous mutant plants, the *AtBolA3* transcript was not detectable by semi-quantitative RT-PCR (Fig 3B). The nearly complete loss of expression of *AtBolA3* was further confirmed by qRT-PCR (Fig 3C). However, it seemed that the loss-of-function of *AtBolA3* did not affect the plants under normal growth condition, as the mutant plants did not exhibit any visible phenotype when grown in soil or in regular hydroponic culture.

### The cytosolic BolA3 is not required for the biogenesis of some typical Fe-S proteins. However, the mutant plants are more tolerant to excess iron

**Fig 4:**
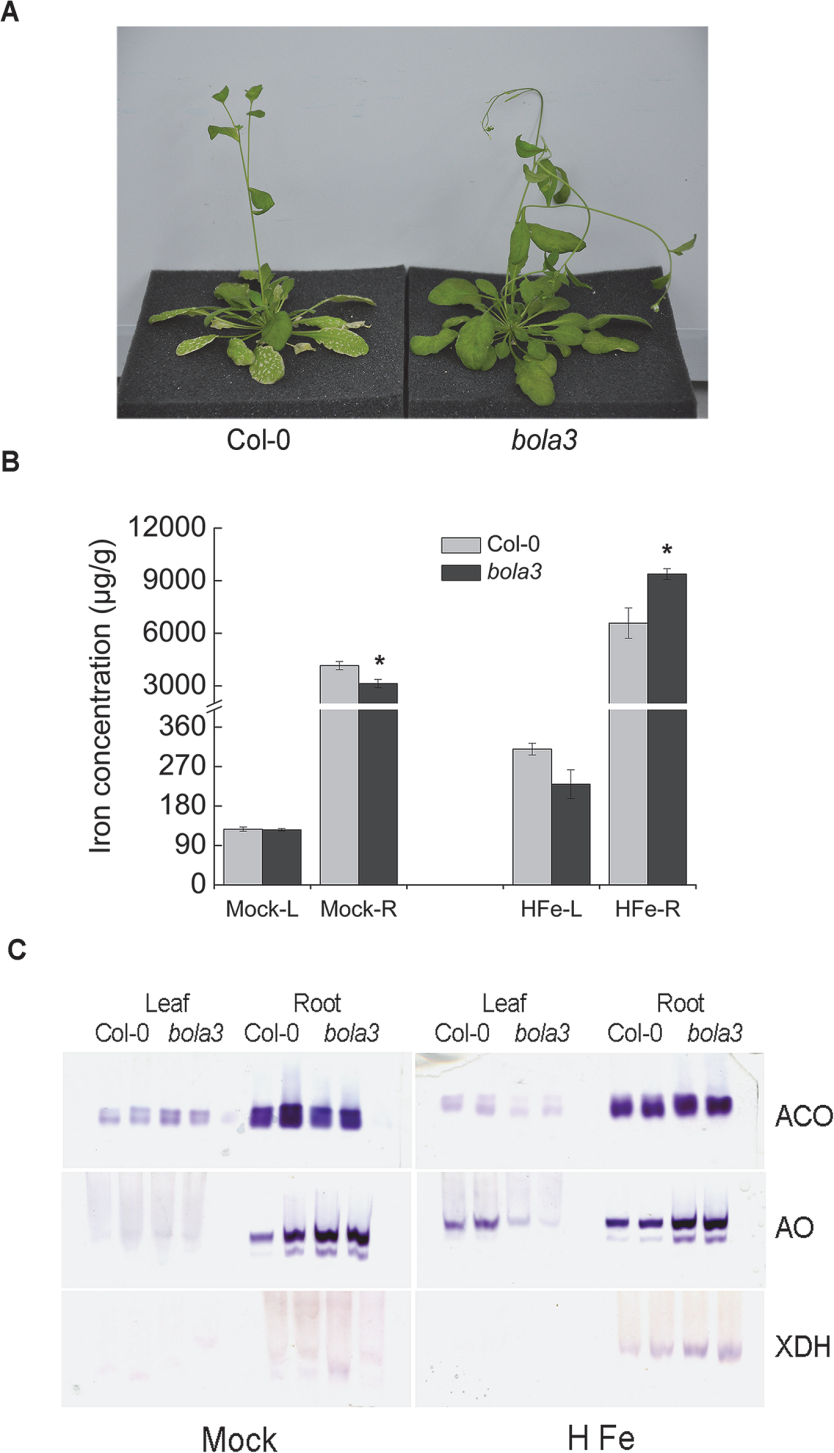
Phenotypes of the *bola3* insertion mutant under excess iron condition. (A) Comparison of the phenotype in the shoot of Col-0 and *bola3* mutant treated with excess iron for four weeks. (B) Iron concentration in the leaf and root of Col-0 and *bola3* mutant plants treated with excess iron for four weeks. L and R: leaf and root. Three biological replicates were used for each measurement. Asterisks indicate significant difference (P<0.05) between Col-0 and *bola3* mutant. The unit of iron concentration was μg of iron/g of dry weight. (C) In-gel activities of aconitase (ACO), aldehyde oxidase (AO) and xanthine dehydrogenase (XDH) in Col-0 and *bola3* mutant plants treated with excess iron for four weeks. Two lanes per genotype in the gels represented two biological replicates.

To investigate whether AtBolA3 is involved in iron metabolism, we treated the 4-week old Col-0 and *bola3* mutant plants in various iron concentrations, including iron deficiency, normal iron, and excess iron, for 4 weeks. Under iron deficiency and normal iron conditions, Col-0 and *bola3* mutant plants grew similarly, and did not exhibit significant difference in main root length and overall growth (not shown). However, under excess iron condition, the growth of mutant plants appeared to be healthier than that of Col-0 plants. The *bola3* mutant plants did not show necrosis in leaves, whereas Col-0 plants showed necrosis, which was initially found on old leaves and eventually extended to all leaves (Fig 4A). In addition, the main root length of mutant plants was 50% longer than that of Col-0 plants (S2 Fig), suggesting that *bola3* mutant plants are more tolerant to excess iron. Next, we measured the iron concentration in leaves and roots of Col-0 and *bola3* mutant plants. Under normal condition, iron concentration in roots was slightly decreased in the mutant plants, whereas iron concentration in leaves was not altered (Fig 4B). By contrast, under excess iron condition, iron concentration in roots of mutant plants was 42% higher than that of Col-0 plants (Fig 4B). To further determine whether AtBolA3 is involved in Fe-S cluster assembly, we performed in-gel activity assays for three typical Fe-S enzymes, including ACO, AO and XDH. There was no significant difference in the Fe-S protein activities between Col-0 and *bola3* mutant plants under normal condition (Fig 4C and S3 Fig), suggesting that AtBolA3 is not required for the biogenesis of some typical Fe-S proteins. By contrast, under excess iron, activities of the Fe-S proteins appeared to be slightly increased in roots of the mutant plants (Fig 4C and S3 Fig), in spite of a slight decrease in leaves. These results together suggested that *bola3* mutant plants were more tolerant to excess iron. In another word, under excess iron condition, AtBolA3 has a repressive role in the iron uptake/storage and Fe-S protein maturation in roots. A function of AtBolA3 in roots is consistent with the result that the expression of *AtBolA3* was high in roots (Fig 2B and 2C).

### The *bola3* mutant plants are more tolerant to the MV-induced oxidative stress

**Fig 5:**
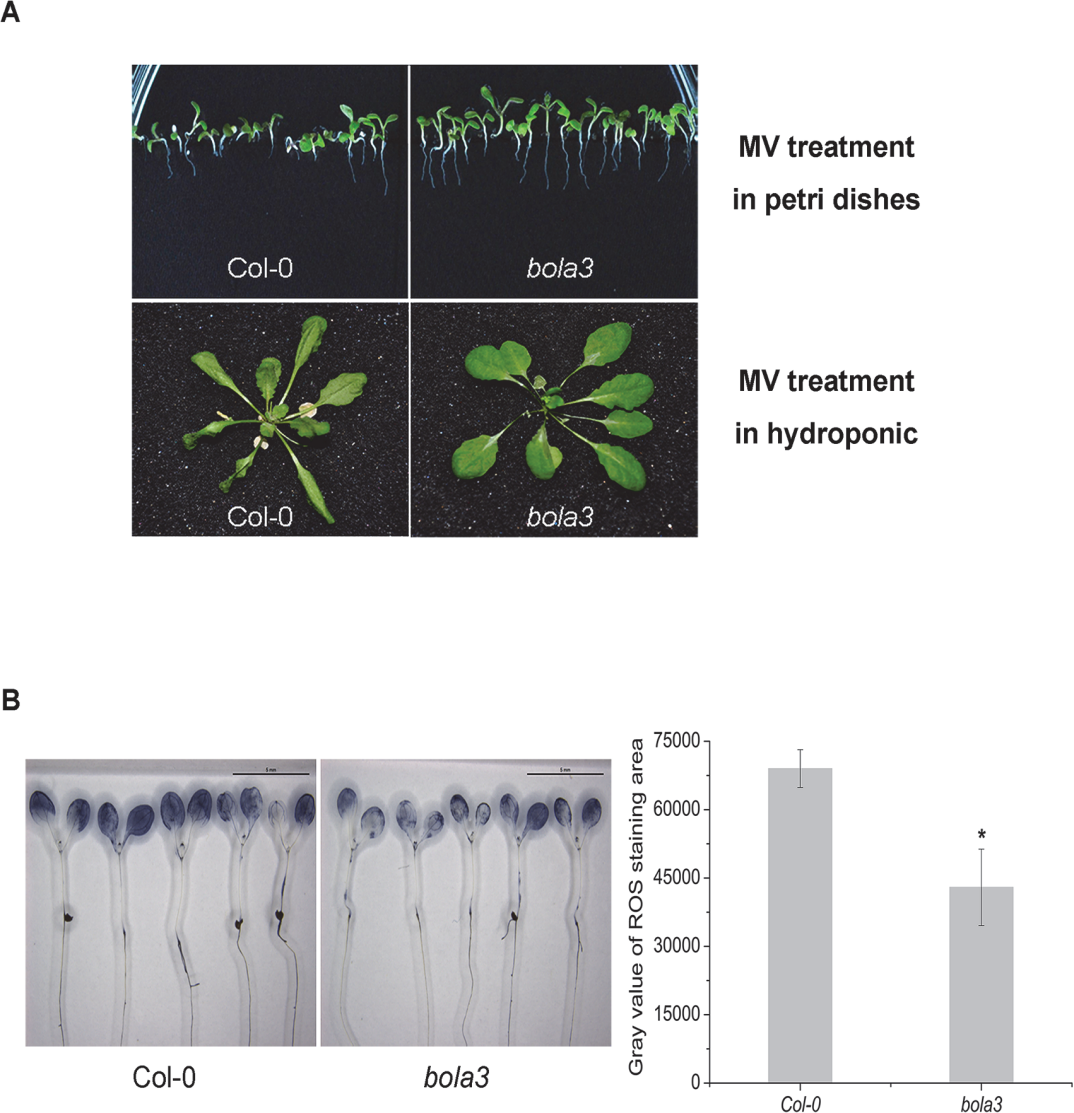
Phenotypes of the *bola3* insertion mutant under the MV-induced oxidative stress. (A) Comparison of the phenotype of Col-0 and *bola3* mutant plants under the MV-induced oxidative stress. Seeds were germinated and grown in plates containing 0.15 μM MV for 2 weeks (top). The 4-week old plants were grown in hydroponic culture containing 50 μM MV for 12 hours (bottom). (B) Superoxide anion accumulation in the one-week-old Col-0 and *bola3* mutant seedlings treated with 2 μM MV for 2 hours. The superoxide staining was quantified with Image J software. Five independent biological replicates were used for each genotype. Asterisk indicates P<0.05.

Several studies have reported that some BolA proteins are involved in the regulation of redox homeostasis [19,22]. To assess a possible phenotype of the *bola3* mutant under oxidative stress, we treated Col-0 and *bola3* plants with methyl viologen (MV) in plates and in hydroponic culture (Fig 5A). In plates, the growth of Col-0 was inhibited by oxidative stress. By contrast, the growth of the *bola3* mutant appeared to be healthier than Col-0, as suggested by a longer root length. In hydroponic culture, the *bola3* mutant did not show necrosis in leaves, whereas Col-0 plants demonstrated remarkable necrosis in leaves, a damage caused by oxidative stress (Fig 5A). The results suggested that the *bola3* mutant plants are more tolerant to the MV-induced oxidative stress.

**Fig 6:**
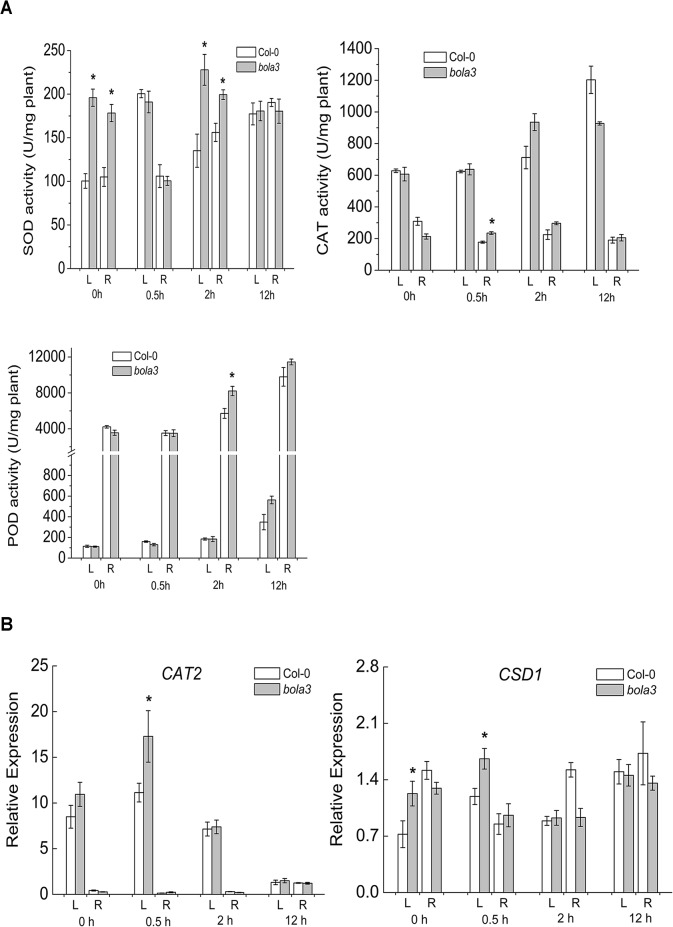
Activities of antioxidant enzymes in the *bola3* insertion mutant under the MV-induced oxidative stress. (A) Activities of antioxidant enzymes, SOD, CAT and POD, in the leaf and root of Col-0 and *bola3* mutant seedlings treated with MV for 0.5 hour, 2 hours and 12 hours. Four biological replicates were used for each measurement. (B) Gene expression of two typical antioxidant enzymes in the leaf and root of Col-0 and *bola3* mutant seedlings treated with MV for 0.5 hour, 2 hours and 12 hours. CAT2: catalase 2; CSD1: Cu/Zn superoxide dismutase 1. Four biological replicates were used for each measurement. Asterisks indicate P<0.05.

As a commonly-used herbicide, MV generates superoxide radicals through the photosynthesis and causes damages to photosystems I and II. We measured the accumulation of superoxide radicals in the MV-treated Col-0 and *bola3* mutant seedlings. The results revealed that the superoxide radical accumulation in *bola3* mutant was significantly lower than that in Col-0 (Fig 5B). Next, we analyzed the activities of antioxidant enzymes (SOD, CAT, and POD) and the expression of two antioxidant defense genes (*CAT2*, *CSD1*). The *CSD1* encodes a Cu/Zn superoxide dismutase, one of the SODs. SOD enzymes scavenge superoxide radicals, whereas CAT and POD enzymes scavenge H_2_O_2._ In comparison with that of Col-0, the SOD enzyme activity was significantly higher in both leaf and root of *bola3* mutant at 0h and 2h upon MV treatment (Fig 6A), and the *CSD1* gene expression was higher in the leaf of *bola3* mutant at both 0h and 0.5h of treatment (Fig 6B). CAT and POD enzyme activities and the *CAT2* gene expression appeared to be slightly increased in mutant (Fig 6A and 6B). These results together suggested that *bola3* mutant plants possess a more potent antioxidant defense, especially the capability to remove superoxide radicals, which may explain that *bola3* mutant plants are more tolerant to the MV-induced oxidative stress. Thereby, AtBolA3 may play a suppressive role in the antioxidant defense.

### The *bola3* mutant plants accumulate more iron in roots under the MV-induced oxidative stress

**Fig 7:**
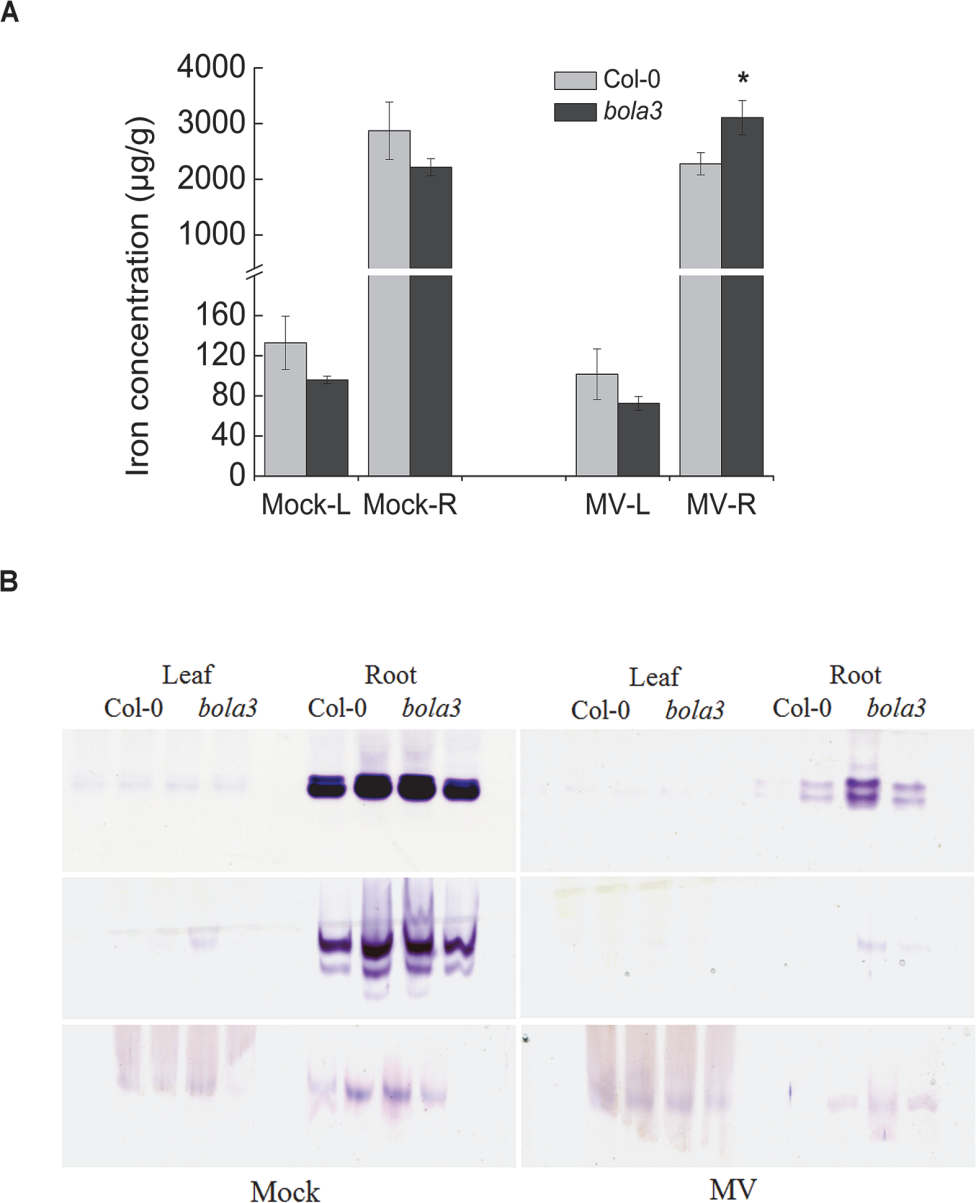
Iron accumulation in roots of *bola3* insertion mutant under the MV-induced oxidative stress. (A) Iron concentration in the leaf and root of Col-0 and *bola3* mutant plants treated with MV for four weeks. L and R: leaf and root. Three biological replicates were used for each measurement. Asterisk indicates P<0.05. The unit of iron concentration was μg of iron/g of dry weight. (B) In-gel activities of ACO, AO and XDH in Col-0 and *bola3* mutant plants treated with MV for four weeks. Two lanes per genotype in the gel represented two biological replicates.

Next, we asked whether iron metabolism is affected by oxidative stress in the *bola3* mutant. We treated Col-0 and *bola3* mutant plants in hydroponic culture containing 2 μM MV and iron at normal concentration, and subsequently measured tissue iron concentrations and activities of three typical Fe-S proteins. Again, *bola3* mutant plants grew better than Col-0 upon MV treatment, as the main root length of the mutant was 20% longer than that of Col-0 (S2B Fig). Interestingly, under the MV-induced oxidative stress, iron concentration in roots of *bola3* mutant was 36% higher than that of Col-0 (Fig 7A). Although Fe-S protein activities were severely impaired by oxidative stress, the remaining activities of Fe-S proteins in roots of *bola3* mutant appeared to be higher than that of Col-0 (Fig 7B and S4 Fig). Taken together, these data suggested that the MV-induced oxidative stress stimulates iron accumulation in roots of the *bola3* mutant.

To confirm that the knockout of *AtBolA3* is the cause of phenotypes, we performed genetic complementation to the mutant by expressing a wild type *AtBolA3* sequence in *bola3* mutant plants. In comparison with Col-0, the resulting complementation plants did not demonstrate significant difference in the phenotype and in root length under either stress (S5A and S5B Fig). Both semi-quantitative RT-PCR and quantitative RT-PCR verified that the *AtBolA3* transgene was normally expressed in the complemented mutant plants (S5C and S5D Fig). The data suggested that the mutation of *AtBolA3* is the cause of phenotypes.

### Interaction of AtBolA3 and AtGRXS17 in cytosol

**Fig 8:**
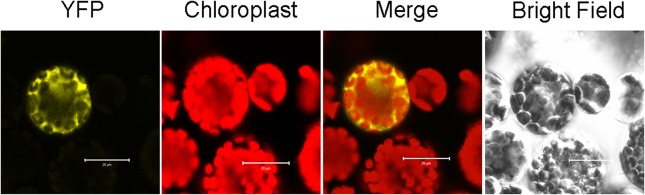
BiFC assay for the interaction of AtBolA3 and AtGRXS17 in Arabidopsis protoplasts. YFP panel is the fluorescence in the Arabidopsis protoplast transfected with BiFC constructs of *AtBolA3* and *AtGRXS17*. Chloroplast panel is the auto-fluorescence of chloroplasts in Arabidopsis protoplast. Merge panel is the merged view of YFP and chloroplast fluorescence.

Numerous studies have shown that a BolA-like protein can complex with a monothiol glutaredoxin of the same subcellular compartment. The bioinformatics data indicated that two candidate proteins, AtGRXS17 (ScGrx3/4 homolog) and aminopeptidase P1 (AtAPP1, ScFra1 homolog), are potential AtBolA3-interacting partners. To confirm the interaction, we performed bimolecular fluorescence complementation (BiFC) analysis. Although co-transfection of *AtBolA3* and *AtPP1* did not generate fluorescence, the BiFC assay for AtBolA3 and AtGRXS17 generated YFP fluorescence (Fig 8), supporting an AtBolA3-AtGRXS17 interaction. And this interaction was found in cytosol.

## Discussion

In this study, we have characterized a homozygous *bola3* mutant line. The *AtBolA3* gene encodes a BolA-like protein in the cytosol of plant cells, and was highly expressed in roots. The mutant plants were more tolerant to some abiotic stresses than wild type. Further analysis revealed that AtBolA3 plays a suppressive role in the tolerance to excess iron and oxidative stress.

Some studies have proposed a function in redox regulation for BolA-like proteins, for instance, the plastid SufE1 and cytosolic BolA of Arabidopsis and the mitochondrial BOLA1 of humans [19,22]. The activity of BolA is dependent on the interacting glutaredoxin in Arabidopsis. The HsBOLA1 may function by increasing the activity of GLRX5 [22]. Our data suggested that the activity of AtBolA3 is not critical under normal conditions, but it is involved in the response to abiotic stresses. To be functional, perhaps AtBolA3 needs to interact with AtGRXS17. Thereby, it raises a possibility that a regulatory complex of GRXS17-BolA3, similar to the Grx3/4-Fra2 complex in yeast, is present in the cytosol of plant cells.

BolA-like proteins always work together with a glutaredoxin through protein-protein interaction, and AtBolA3 seems to be a repressor in the response to abiotic stresses. Hence, we propose that AtBolA3 is a repressor or a negative regulator to AtGRXS17. The later protein plays a positive role in the redox homeostasis and perhaps in iron metabolism. Study on a T-DNA insertion mutant has confirmed that AtGRXS17 plays a critical role in redox homeostasis. The *AtGRXS17* gene expression is induced in response to high temperature. In heat shock, the *AtGRXS17* loss-of-function plants display severe growth defects and increased ROS levels [32]. Overexpression of *AtGRXS17* in tomato plants could increase activity of catalase and expression of heat shock proteins, reduce hydrogen peroxide accumulation, and thereby reduce oxidative damages under heat shock and oxidative stress [33]. Studies on the GRXS17 homologs in animals reveal similar results. The mammalian homolog is called Grx3, TXNL2 or PICOT. The *Grx3* in mice is induced by various oxidants, and Grx3 has a conserved function in protecting cells against oxidative stress [34]. TXNL2 plays an important role in antagonizing oxidative stress in cancer cells. *TXNL2* is found to be overexpressed in cancer cells, thereby stimulating the growth of cancer cells. Knockdown of *TXNL2* increases the intracellular ROS levels, thereby inhibiting the proliferation, survival, and invasion of cancer cells. The data suggest that TXNL2 could be a target for treatment of cancer [35]. It would be of interest to examine whether BolA2, the interaction partner of TXNL2, is a repressor or a negative regulator to the activity of TXNL2. Moreover, it might be possible to employ BolA2 to shrink tumors by inhibiting TXNL2.

In the highly conserved BolA motif, a very C-terminal histidine residue is conserved in all BolA proteins. As demonstrated for yeast BolA2/Fra2 and human BolA2, this histidine residue is required for ligation of a 2Fe-2S cluster, thereby bridging the BolA with a monothiol glutaredoxin [17,21]. Numerous studies have indicated that all CGFS-type monothiol glutaredoxins can assemble Fe-S clusters [36,37,38]. We predict that the AtBolA3-AtGRXS17 protein complex could assemble a 2Fe-2S cluster in it. Since AtBolA3 is not required for Fe-S cluster assembly, the bridging 2Fe-2S cluster is likely used as a redox sensor, or a sensor for the cellular iron status. The AtBolA3-AtGRXS17 complex has to acquire its Fe-S cluster from the cytosolic iron-sulfur cluster assembly (CIA) machinery. Interestingly, a phenotype of resistance to the MV-induced oxidative stress is also found on the insertion mutant of *NAR1*, which encodes a member of the CIA machinery in Arabidopsis [39].

Excess iron generates hydrogen peroxide and hydroxyl radicals via the Fenton reaction, whereas MV generates superoxide radicals. These are different ROS species, which may impair iron homeostasis in different ways. Indeed, the gene expression of *AtIRT1*, encoding a principal iron transporter in roots, was not altered by excess iron, but was completed abolished by MV treatment (S6 Fig). Iron concentration in roots of *bola3* mutant was enhanced by MV treatment. The mechanism remains unknown. Perhaps alternative iron transporters are expressed in roots, which are not suppressed by the oxidative stress. MV is a potent oxidant, and the MV-induced ROS severely impaired the Fe-S protein activities (Fig 7B). The Fe-S protein activities in *bola3* mutant appeared to be higher than that of Col-0 in MV treatment (Fig 7B and S4 Fig). It is probably because that the mutant possessed a potent antioxidant defense, which could protect the redox-sensitive Fe-S centers. Nevertheless, the results that more iron was accumulated in roots of *bola3* mutant under both excess iron and oxidative stress strongly suggest an involvement of AtBolA3 in the regulation of normal iron homeostasis in response to abiotic stresses.

BolA-like proteins located in various subcellular compartments may have different functions. Although the cytosolic BolA is not required for Fe-S cluster assembly, BolA proteins in subcellular organelles could be involved in Fe-S synthesis. For example, the human mitochondrial HsBolA3 plays an essential role in the production of Fe-S centers for the normal maturation of 2-oxoacid dehydrogenases and respiratory chain complexes [23]. Monothiol glutaredoxins (GRX) are thought to be implicated in Fe-S cluster assembly in organelles, for instance the mitochondrial HsGLRX5 and chloroplastic AtGRXS14/16, whereas cytosolic monothiol glutaredoxins are thought to play critical roles in redox homeostasis, for instance the HsPICOT and AtGRXS17. Because a universal BolA-GRX interaction has been identified in numerous eukaryotic species, it is likely that a BolA protein is functional by interacting with a GRX, and even by regulating the GRX.

The knockout/knockdown of *AtBolA3* seems to offer the plants an advantage to survive abiotic stresses, including excess iron and oxidative stress. One may ask whether *AtBolA3* is a useless gene, and that a super-crop might be bred by eliminating the *AtBolA3* gene. However, the loss of *AtBolA3* could potentially have adverse effects on plants, for instance a compromised defense to drought and disease, which remain to be investigated.

## Supporting Information

S1 Fig Coomassie blue staining of SDS-PAGE gels as an indication of equal loading. (TIF)

S2 Fig Phenotypes of *bola3* insertion mutant under excess iron and the MV-induced oxidative stress. (A) Plant phenotype and main root length of Col-0 and *bola3* mutant exposed to excess iron. (B) Plant phenotype and main root length of Col-0 and *bola3* mutant plants treated with MV. At least four biological replicates were used for each measurement. Asterisks indicate P<0.05.
(TIF)

S3 Fig Quantitative determination of ACO, AO and XDH enzyme in-gel activities under excess iron treatment using Image J. (A): Quantitative determination of ACO activities; (B): Quantitative determination of AO activities; (C): Quantitative determination of XDH activities.
(TIF)

S4 Fig Quantitative determination of ACO, AO and XDH enzyme in-gel activities under the MV-induced oxidative stress using Image J. (A): Quantitative determination of ACO activities; (B): Quantitative determination of AO activities; (C): Quantitative determination of XDH activities.
(TIF)

S5 Fig Complementation of phenotypes by expressing a WT sequence of *AtBolA3* in the mutant plants. (A) Growth of Col-0 and the *bola3* mutant transformed with construct of *AtBolA3* driven by 35S promoter (*AtBolA3*+*bola3*) under excess iron and MV treatment. (B) Root length of Col-0 and *AtBolA3*+*bola3* seedlings under excess iron and MV-induced oxidative stress. At least ten seedlings were used for each measurement. NS: non-significant. (C) Analysis of *AtBolA3* gene expression in Col-0 and *AtBolA3*+*bola3* seedlings by semi-qRT-PCR. (D) Analysis of *AtBolA3* gene expression in Col-0 and *AtBolA3*+*bola3* seedlings by qRT-PCR.
(TIF)

S6 Fig Expression levels of *AtIRT1* in the leaf and root of Col-0 and *bola3* mutant seedlings under excess iron and MV treatment. (TIF)

S1 Table Primers for this study. (DOC)
