# Supplementary figures and images for: Cytosolic BolA Plays a Repressive Role in the Tolerance against Excess Iron and MV-Induced Oxidative Stress in Plants
Source: PLoS One. 2015 Apr 30;10(4):e0124887. doi: 10.1371/journal.pone.0124887 (PMC4415784; doi:10.1371/journal.pone.0124887)

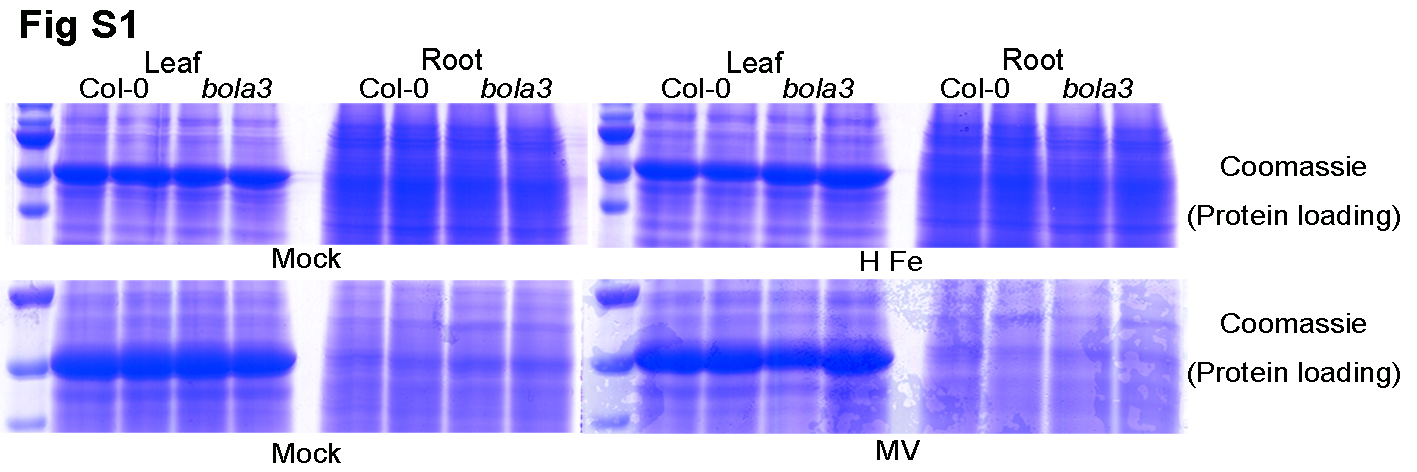

Supplement: S1 Fig — (TIF) [file pone.0124887.s001.tif]

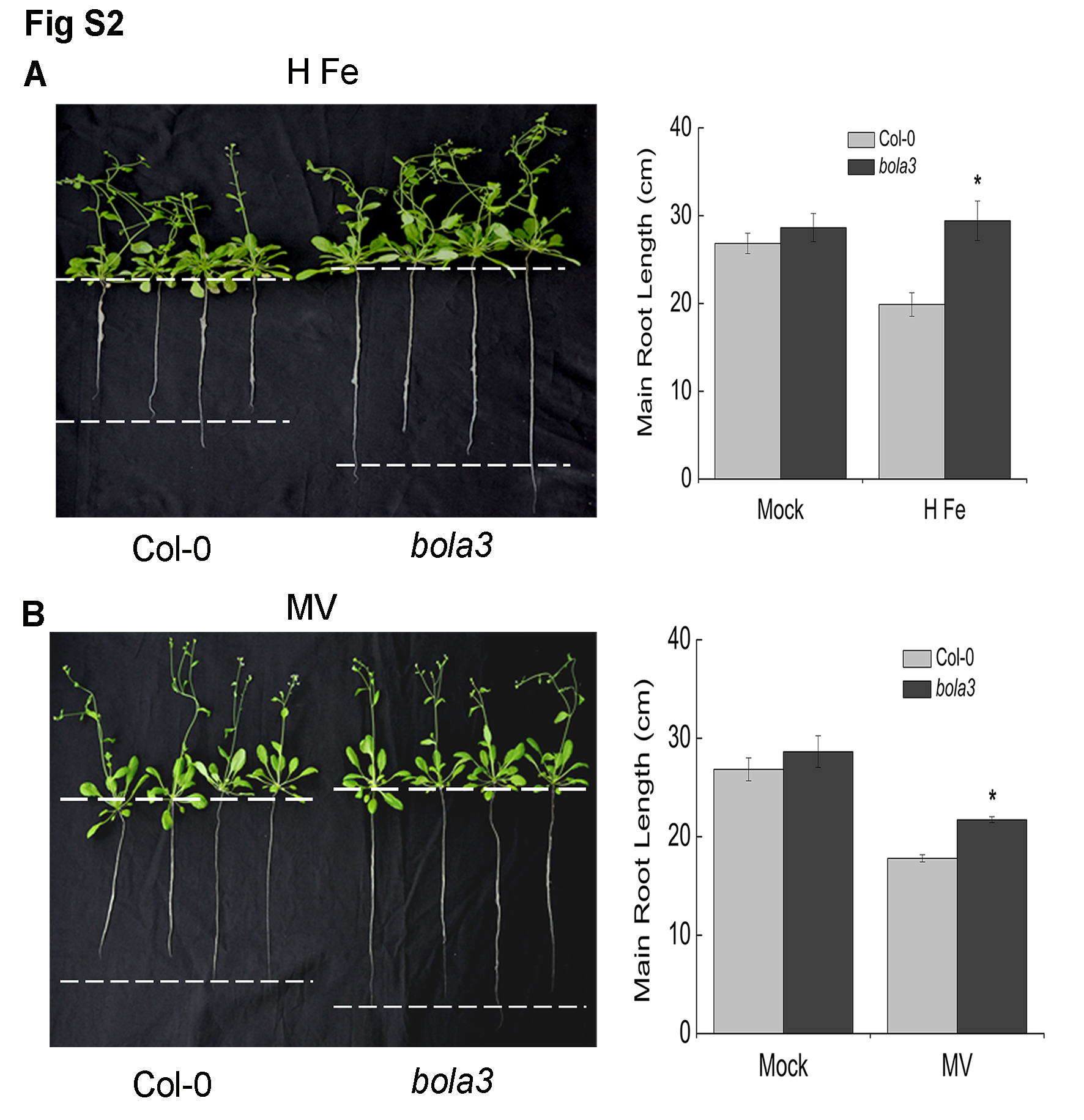

Supplement: S2 Fig — (A) Plant phenotype and main root length of Col-0 and bola3 mutant exposed to excess iron. (B) Plant phenotype and main root length of Col-0 and bola3 mutant plants treated with MV. At least four biological replicates were used for each measurement. Asterisks indicate P<0.05. (TIF) [file pone.0124887.s002.tif]

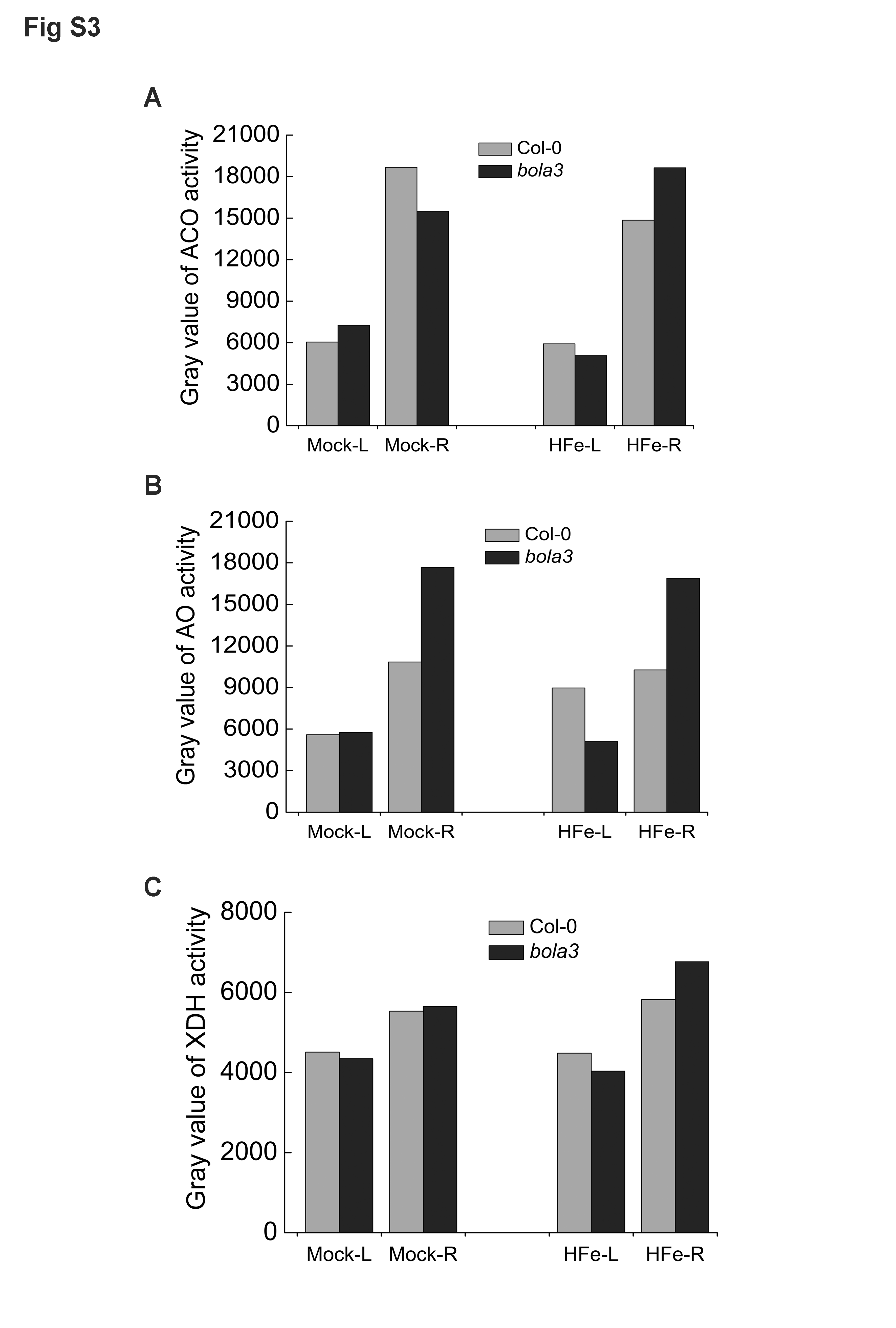

Supplement: S3 Fig — (A): Quantitative determination of ACO activities; (B): Quantitative determination of AO activities; (C): Quantitative determination of XDH activities. (TIF) [file pone.0124887.s003.tif]

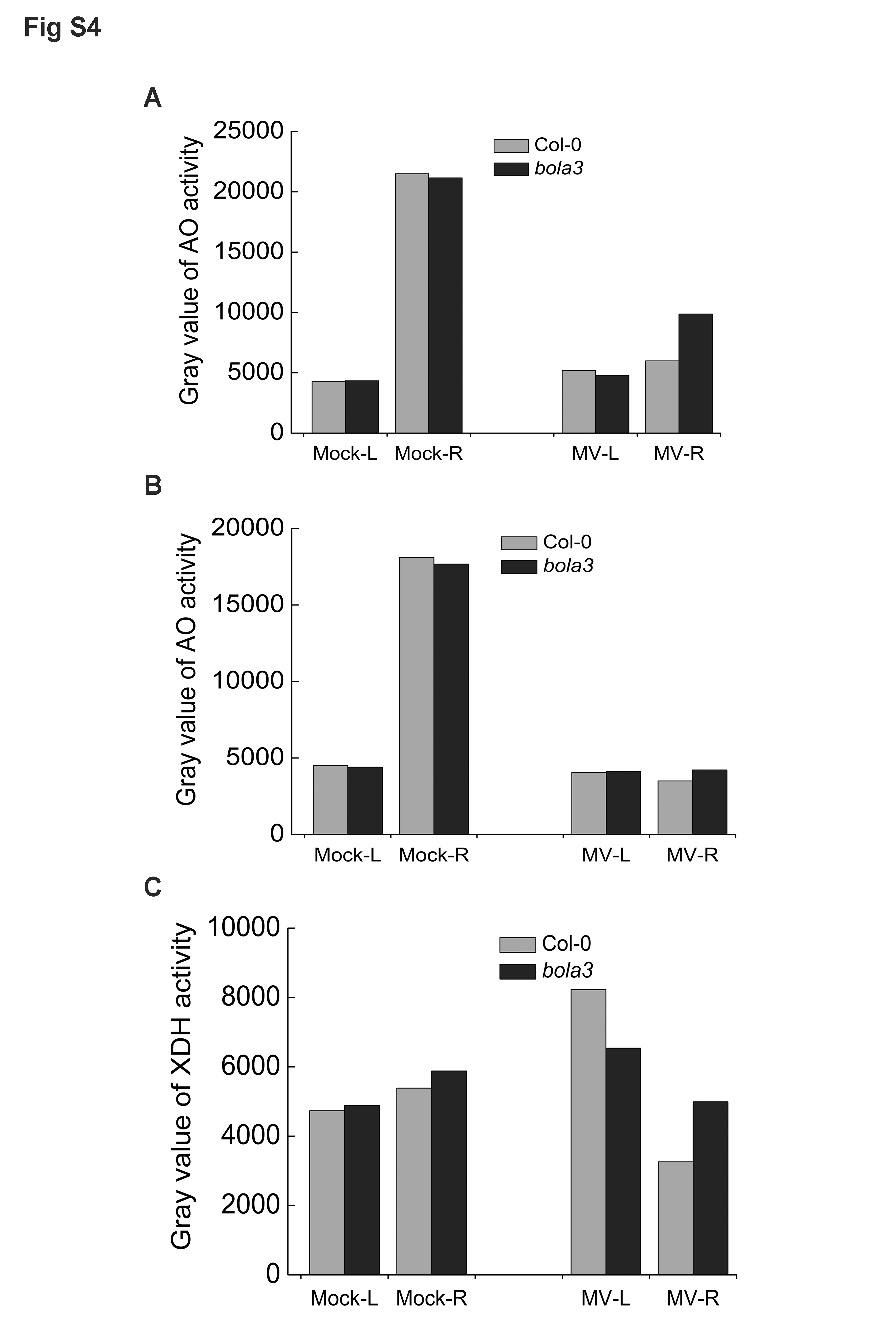

Supplement: S4 Fig — (A): Quantitative determination of ACO activities; (B): Quantitative determination of AO activities; (C): Quantitative determination of XDH activities. (TIF) [file pone.0124887.s004.tif]

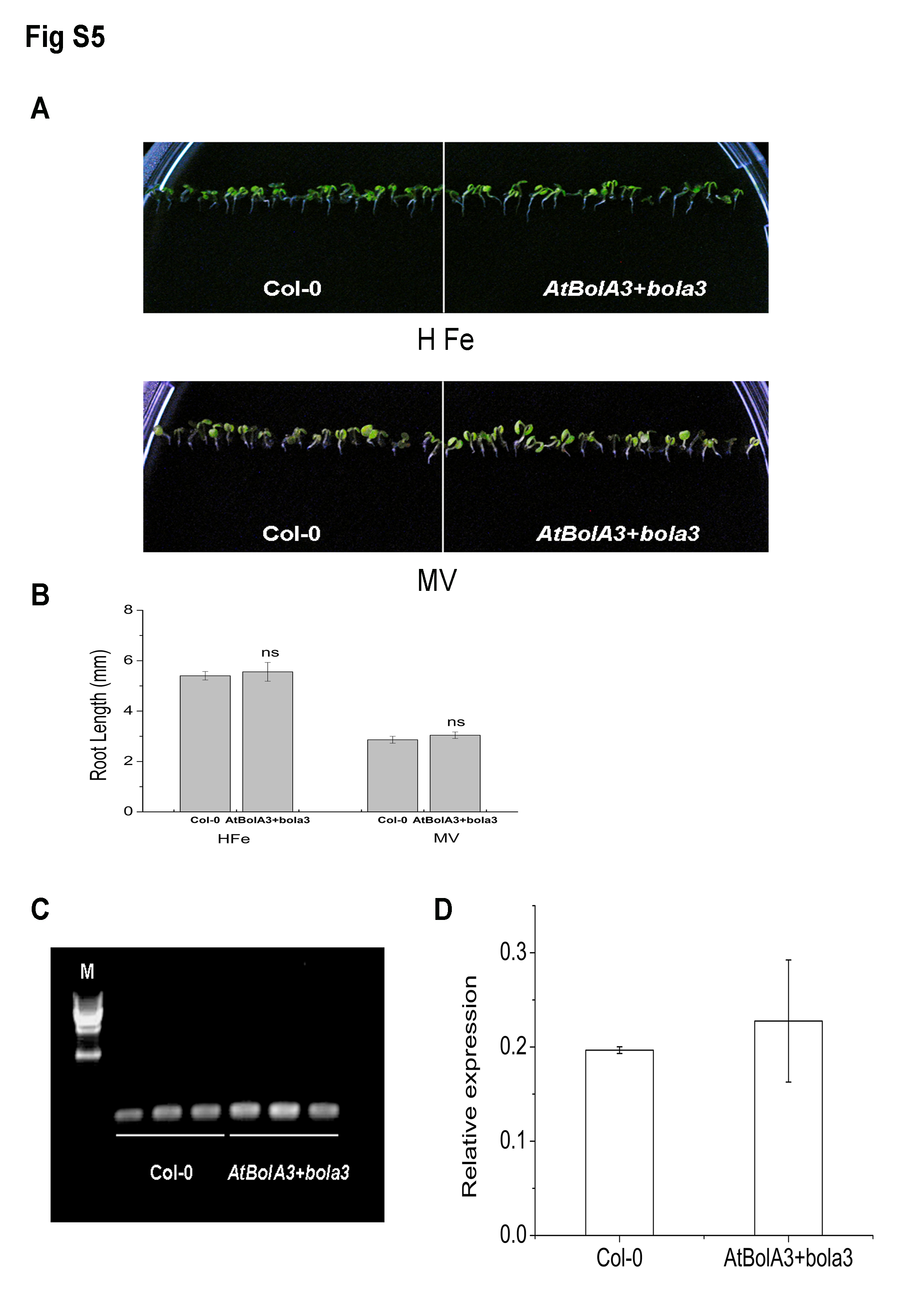

Supplement: S5 Fig — (A) Growth of Col-0 and the bola3 mutant transformed with construct of AtBolA3 driven by 35S promoter (AtBolA3+bola3) under excess iron and MV treatment. (B) Root length of Col-0 and AtBolA3+bola3 seedlings under excess iron and MV-induced oxidative stress. At least ten seedlings were used for each measurement. NS: non-significant. (C) Analysis of AtBolA3 gene expression in Col-0 and AtBolA3+bola3 seedlings by semi-qRT-PCR. (D) Analysis of AtBolA3 gene expression in Col-0 and AtBolA3+bola3 seedlings by qRT-PCR. (TIF) [file pone.0124887.s005.tif]

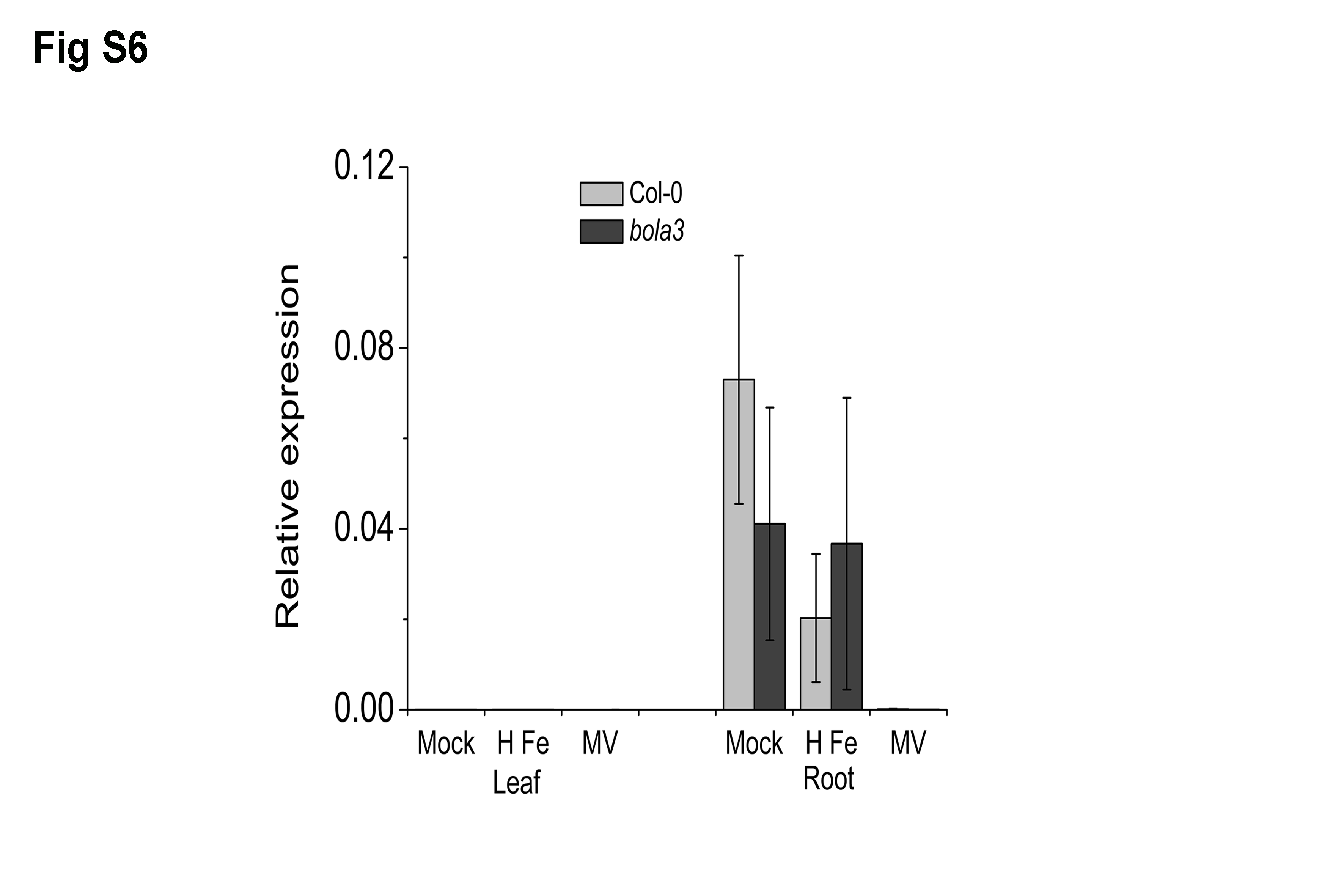

Supplement: S6 Fig — (TIF) [file pone.0124887.s006.tif]
