# Supplementary material for: Cytosolic BolA Plays a Repressive Role in the Tolerance against Excess Iron and MV-Induced Oxidative Stress in Plants
Source: PLoS One. 2015 Apr 30;10(4):e0124887. doi: 10.1371/journal.pone.0124887 (PMC4415784; doi:10.1371/journal.pone.0124887)
Supplement: S1 Table — (DOC) [file pone.0124887.s007.doc]

**S1 Table. Primers for this study.**

| **Primer name** | **sequence** |
| --- | --- |
| **Primer sequences for subcelluar localization** |  |
| AtBo1A1-gfp-F | 5’-ACGCGTCGACATGGCAGCAGCGATGTCTTC-3’ |
| AtBo1A1-gfp-R | 5’-CCCGAGCTCGAAACCTCAGCAGGAGTCTTTGC-3’ |
| AtBo1A2-gfp-F | 5’-ACGCGTCGACATGTTTTCATCTTCGATTAG-3’ |
| AtBo1A2-gfp-R | 5’-CATGCCATGGAGTCTTTAGAAGGAGACTCAG-3’ |
| AtBo1A3-gfp-F | 5’-ACGCGTCGACATGGTGACGAAGGAGCAAGT-3’ |
| AtBo1A3-gfp-R | 5’-CCCGAGCTCGAGGCATCTTTGGTTAAGGTTGC-3’ |
| AtBo1A4-gfp-F | 5’-ACGCGTCGACATGGCGCAGACATTAATGGC-3’ |
| AtBo1A4-gfp-R | 5’-CCCGAGCTCGAAACTTCAGAAGGAGTCTTTG-3’ |
|  |  |
| **Primer sequences for promoter analysis** |  |
| *AtBo1A3prom*-GUS-F | 5’-CCCAAGCTT CCAAATCAGGAGGGAAG-3’ |
| *AtBo1A3prom*-GUS-R | 5’-AACTGCAG ATCCCTAATTACTGTAC-3’ |
|  |  |
| **Primer sequences for complementation** |  |
| *AtBo1A3*-CDS-F | 5’-GCTCTAGAGATGGTGACGAAGGAGC-3’ |
| *AtBo1A3*-CDS-R | 5’-GCGGAT CCCAGGCATCTTTGG-3’ |

| **Primer name** | **sequence** |
| --- | --- |
| **Primer sequences for mutant identification** |  |
| bola3-LP | 5’- AAATTTAGCCAATCCCATTGG -3’ |
| bola3-RP | 5’- GTCGAAGCTTCATTGACTTCG -3’ |
| LBb1.3 | 5’-ATTTTGCCGATTTCGGAAC-3’ |
|  |  |
| **Primer sequences for qRT-PCR** |  |
| *AtBolA3*-F | 5’-AGGCATCGTATGGTGAATG-3’ |
| *AtBolA3*-R | 5’-GGTTAAGGTTGCAGAGTCTTG-3’ |
| *AtCAT2*-F | 5’-TCCGCCTGCTGTCTGTTCTG-3’ |
| *AtCAT2*-R | 5’-TGGGTCGGATAGGGCATCAA-3’ |
| *AtCSD1*-F | 5’-TGATGGAACTGCCACCTTCACA-3’ |
| *AtCSD1*-R | 5’-ATGGCCTCCCTTTCCGAGGT-3’ |
| *AtIRT1*-F | 5’-GCTCTTGCGATACCGAGTCA-3’ |
| *AtIRT1*-R | 5’-GATACAACCGCCAAGACCCA-3’ |
| *AtUBQ11*-F | 5’-GCAGATTTTCGTTAAAACC-3’ |
| *AtUBQ11*-R | 5’-CCAAAGTTCTGCCGTCC-3’ |
|  |  |
| **Primer sequences for BiFC** |  |
| *AtBolA3*-BiFC-C-F | 5’-ATCGAGATCTATGGTGACGAAGGAGCAAGT-3’ |
| *AtBolA3*-BiFC-C-R | 5’-ATCGGGATCCtcaGGCATCTTTGGTTAAGGTTGC-3’ |
| *AtGRX17*-BiFC-C-F | 5’-ATCGGAATTCcATGAGCGGTACGGTGAAGG-3’ |
| *AtGRX17*-BiFC-C-R | 5’-ATCGGGATCCttaCTCGGATAGAGTTGCTTTGAGA-3’ |
| *AtAPP1-BiFC-C-F* | 5’-ATCGAGATCTcgATGTCCGAGATTCTCTCTTCG-3’ |
| *AtAPP1-BiFC-C-R* | 5’-ATCGGGTACCtcaAGCGGATACACTTACAGGTTCG-3’ |
